# Mathematical pharmacodynamic modeling for antimicrobial assessment of ceftazidime/colistin versus gentamicin/meropenem combinations against carbapenem-resistant *Pseudomonas aeruginosa* biofilm

**DOI:** 10.1186/s12941-023-00597-9

**Published:** 2023-07-02

**Authors:** Mona Shaban E. M. Badawy, Walid F. Elkhatib, Rania I. Shebl

**Affiliations:** 1 Department of Microbiology and Immunology, Faculty of Pharmacy (Girls), El-Azhar University, Cairo, Egypt; 2 Microbiology and Immunology Department, Faculty of Pharmacy, Ain Shams University, African Union Organization St., Abbassia, Cairo 11566 Egypt; 3 Department of Microbiology & Immunology, Faculty of Pharmacy, Galala University, New Galala City, Suez, Egypt; 4 Department of Microbiology and Immunology, Faculty of Pharmacy, Ahram Canadian University, 6th October city, 4th industrial zone, Giza, 12451 Egypt

**Keywords:** Pharmacodynamic, *Pseudomonas aeruginosa*, Ceftazidime, Colistin, Meropenem, Gentamicin, Biofilm

## Abstract

**Background:**

Carbapenem-resistant *Pseudomonas aeruginosa* (CRPA) represents an escalating healthcare hazard with high mortality worldwide, especially in presence of biofilm. The current study aimed to evaluate the anti-biofilm potentials of ceftazidime, colistin, gentamicin, and meropenem alone and in combinations against biofilm-forming CRPA.

**Methods:**

Biofilm killing and checkerboard assay were performed to detect the effectiveness of combined antibiotics against biofilms and planktonic cells, respectively. The bacterial bioburden retrieved from the established biofilms following treatment with combined antibiotics was utilized to construct a three-dimensional response surface plot. A sigmoidal maximum effect model was applied to determine the pharmacodynamic parameters (maximal effect, median effective concentration, and Hill factor) of each antibiotic to create a mathematical three-dimensional response surface plot.

**Results:**

Data revealed statistically significant (p < 0.05) superior anti-biofilm potential in the case of colistin followed by a lower effect in the case of gentamicin and meropenem, while ceftazidime exhibited the least anti-biofilm activity. The fractional inhibitory concentration index (FICI ≤ 0.5) indicated synergism following treatment with the combined antibiotics. An elevated anti-biofilm activity was recorded in the case of gentamicin/meropenem compared to ceftazidime/colistin. Synergistic anti-biofilm potentials were also detected via the simulated pharmacodynamic modeling, with higher anti-biofilm activity in the case of the in vitro observation compared to the simulated anti-biofilm profile.

**Conclusions:**

The present study highlighted the synergistic potentials of the tested antibiotic combinations against *P. aeruginosa* biofilms and the importance of the mathematical pharmacodynamic modeling in investigating the efficacy of antibiotics in combination as an effective strategy for successful antibiotic therapy to tackle the extensively growing resistance to the currently available antibiotics.

## Background

*Pseudomonas aeruginosa* is regarded as one of the leading causes of hospital-acquired and hazardous infections, especially in immunocompromised patients. The global incidence of multiple drug-resistant *P. aeruginosa* (MDR-PA) infections is increasing, including carbapenem-resistant *P. aeruginosa* (CRPA) strains that are particularly difficult to treat [1]. CRPA is considered one of the initial priority pathogens for the investigation and development of new antibiotics as well as infection control approaches. MDR-PA infections which are resistant to carbapenem are also associated with high mortality rates of up to 61% [2]. For patients with *P. aeruginosa* infections, previous studies showed that applying an empirically designed combined antibiotic approach was more effective than using monotherapy, especially for critically ill and neutropenic patients. Despite the lack of strong randomized controlled studies demonstrating the advantage of antimicrobial combinations, several international guidelines addressing optimal antimicrobial efficacy suggested treating invasive CRPA infections with combination therapy [3].

The management of infections caused by *P. aeruginosa* is considered an obstacle not only as a result of the intrinsic or genetically developed resistance but also due to virulence factors such as biofilm formation [4] as well as its ability to adhere to surfaces [5]. Bacterial biofilm is a surface-associated layer of microbial cells with self-produced extracellular polymeric materials that allow bacteria to survive in harsh environments and then detach to colonize other habitats. Biofilms are typically inherently resistant to high concentrations of antimicrobials; thus, their treatment is almost difficult and costly. Biofilms are also a major cause of illness and mortality as they could be found on many surfaces including living cells in addition to indwelling medical devices [6].

Biofilm removal normally necessitates higher and extended antibiotic therapy. However, this frequently fails to eliminate biofilm-associated infections [7]. Currently, there are limited antibiotic options to control infections with antibiotic-resistant and biofilm-forming *P. aeruginosa*. Clinicians may become obligated to describe the currently available antimicrobials irrespective of their low efficiency or their side effects. Nowadays, there is a reappearing hope for the successful treatment of *P. aeruginosa* infections via the application of combined antimicrobial therapy, which is categorized as an extremely powerful tool for controlling infections associated with *P. aeruginosa* biofilms [4]*.* Hence, the purpose of the present study is to explore the anti-biofilm potentials of different antibiotics alone as well as in combinations against CRPA clinical isolates using mathematical pharmacodynamic modeling.

## Materials and methods

### Bacterial isolates and antimicrobial agents

Two previously isolated and identified *P. aeruginosa* clinical isolates (CRPA-45 and CRPA-47) [8] were selected to be employed in the current study. The selection was performed based on that each isolate is resistant to carbapenem and produces biofilm. Confirming biofilm formation was carried out according to Ruchi et al. [9] post 24 h of allowing biofilm production using the microtiter plate method. The formed biofilms were stained with crystal violet and evaluated against *P. aeruginosa* PAO1 (ATCC 15692) standard strain [9]. *P. aeruginosa* clinical isolates were cultured two times on Tryptic Soy Broth (TSB) supplemented with 1% glucose [10] and incubated overnight at 37 °C before examining the anti-biofilm potentials. Bacterial inoculums were diluted in Mueller Hinton broth (MHB) to match the absorbance of 0.5 McFarland standard, which is corresponding to 1.5 X 10^8^ colony forming unit (CFU) per milliliter. Ceftazidime, colistin, gentamicin, and meropenem (Sigma-Aldrich, Saint Louis, MO, USA) were dissolved in distilled water to prepare stocks at an initial concentration of 1024 µg/ml and stored at −80 °C.

### Minimum inhibitory concentrations (MICs)

Antibiotic susceptibility of the planktonic *P. aeruginosa* clinical isolates was carried out using broth microdilution method according to the Clinical and Laboratory Standard Institute (CLSI) guidelines [11]. Briefly, ninety-six well polystyrene microtiter plates containing double-fold serially diluted antibiotics were further inoculated with the bacterial isolates at a final count of 5 × 10^5^ CFU/ml. Positive control wells were inoculated with MHB medium instead of antibiotics, whereas wells free from bacterial inoculums served as a negative control. Post overnight incubation at 37 °C, the lowest concentration of each antibiotic that showed no observable growth was considered as the MIC. MBC was determined via inoculating 20 µl out of wells showing no growth on agar plates followed by incubation at 37 °C for 24 h. Minimum bactericidal concentration (MBC) is the minimum antibiotic concentration that showed no obvious growth [12].

### Biofilm susceptibility testing

#### Biofilm formation

Bacterial inoculums matching 0.5 MacFarland were further diluted a hundred times using TSB and inoculated as 200 µl/well in 96-well microtiter plates. Negative control wells were inoculated with broth only to check for sterility. The plates were overnight incubated, and the media were gently discarded followed by washing the plates two times with saline [9].

#### Minimum biofilm inhibitory concentration

Double-fold serially diluted antibiotics in MHB were inoculated as 100 µl/well into the plates with established biofilms and overnight incubated. Minimum biofilm inhibitory concentration (MBIC) is recorded as the minimum antibiotic concentration showing no visible growth, where it is the minimum concentration that prevented the release of planktonic cells out of the bacterial biofilm [13].

#### Minimum biofilm bactericidal concentration

Minimizing the carryover of the antibiotic was carried out via transferring 10 µl out of wells showing no apparent growth to plates previously inoculated with 90 µl/well TSB. Post overnight incubation, the minimum biofilm bactericidal concentration** (**MBBC) was recorded as the minimum antibiotic concentration inhibiting an observable bacterial growth. The existence of bacterial growth indicates the ability of the planktonic bacteria to regrow out of the viable biofilms, thus the MBBC value is regarded as the minimum antibiotic concentration where the bacteria fail to retain their ability to grow [14].

### Biofilm killing assay

#### Anti-biofilm of a single antibiotic

*P. aeruginosa* isolates were prepared as 1.5 × 10^6^ CFU/ml in TSB and inoculated as 0.5 ml in 1.5 ml polypropylene tubes followed by overnight incubation in an aerobic environment without shaking. The tubes were rinsed with normal saline after careful aspiration of the supernatant [9]. Tubes with the previously established *P. aeruginosa* biofilms were treated with four antibiotics (ceftazidime, colistin, gentamicin, and meropenem) in an increasing concentration corresponding to 0.25, 0.5, 1, 2, 4, 16, 32, and 64 times MBIC. Untreated control tubes were also considered. All tubes were overnight incubated at 37 °C followed by aspiration of the supernatant containing planktonic cells. Tubes were rinsed gently with normal saline, sonicated for 3 min at low intensity (10%), and vortexed for 60 s to allow the dispersion of the bacterial cells out of their biofilms without decreasing the viability of the dislodged bacterial cells [15]. The total biofilm-embedded bacterial load was determined by aspirating 100 µl from each tube followed by ten-fold serial dilution of the aspirated samples and plating onto Mueller Hinton Agar (MHA) plates. The logarithmic mean of the bacterial burden was plotted against different antibiotic concentrations in a sigmoidal Emax curve. The Hill factor (H) of each antibiotic was calculated. In the meantime, the effective concentration of each antimicrobial agent that caused 50% of the maximum effect (EC_50_) was calculated using the data retrieved from the total bacterial burden following treatment with a single antibiotic [16].

#### Fractional inhibitory concentration index

The micro broth checkerboard technique was applied to test the effect of antibiotics in combination against planktonic cells [17]. In brief, each 96-well microtiter plate was inoculated with two-fold serially diluted two antibiotics ranging from 1/8 MIC to 2 MIC, taking into consideration that one antibiotic is inoculated in the columns and the other is inoculated in rows. The bacterial isolates were then inoculated as 5 × 10^5^ CFU/ml. Plates were overnight incubated under static conditions and observed visually. The following equation was applied to calculate the fractional inhibitory concentration index (FICI): FICI = (MIC of the first antibiotic in combination/MIC of the first antibiotic alone) + (MIC of the second antibiotic in combination/MIC of the second antibiotic alone). The combination between the tested antibiotics was categorized as synergism if the calculated FICI was less than or equal to half and indifferent when the FICI value was in the range between values greater than 0.5 and less than 4.0. While the relation between the 2 antibiotics was considered antagonism in case of obtaining a FICI value of more than or equal to 4.0 [18].

### Anti-biofilm of antibiotic combinations and pharmacodynamic modeling

The anti-biofilm activity of twenty-five antibiotic combinations was examined against *P. aeruginosa* biofilm. The entire bacterial load was recovered from the established biofilms as previously described and counted following each treatment with antibiotics in combination as well as in the case of untreated positive control. A three-dimensional response surface plot was created based on the obtained data. Regarding the pharmacodynamic modeling, another simulated three-dimensional response surface was obtained depending on calculating the summative effect of the combined antibiotics under an assumption of null interaction using the following equation [16]. All the three-dimensional response surface plots were presented with the aid of OriginPro 2018 software (OriginLab Corporation, Northampton, USA).

$$Log\,CFU/ml = E_{0} -\left\{ {\left[ {\frac{{{\mathbf{E}}_{{\mathbf{max} \,{\mathbf{A}}}} \cdot \,{\mathbf{C}}_{{\mathbf{A}}}^{{{\mathbf{HA}}}} }}{{{\mathbf{C}}_{{\mathbf{A}}}^{{{\mathbf{HA}}}} + {\mathbf{C}}_{{{\mathbf{50A}}}}^{{{\mathbf{HA}}}} }}} \right]+\left[ {\frac{{{\text{E}}_{{\max \,{\text{B}}}} \cdot \,{\text{C}}_{{\text{B}}}^{{{\text{HB}}}} }}{{{\text{C}}_{{\text{B}}}^{{{\text{HB}}}} +{\text{C}}_{{{\text{50B}}}}^{{{\text{HB}}}} }}} \right]} \right\}$$

where E_0_ indicates the average bacterial count in the positive control, E_max A_ & E_max B_ represent the maximum inhibitory potentials of antibiotics A & B, respectively. C_A_ & C_B_ refer to antibiotic A & antibiotic B concentrations, respectively. C_50A_ & C_50B_ indicate the concentrations of both antibiotics yielding 50% of the optimum effect. HA and HB refer to the Hill factors for both antibiotics, respectively.

### Statistical analysis

All tests were carried out in independent triplicates and the results were expressed in terms of the mean ± standard deviation (SD). Statistical analysis was performed via statistical package for social sciences SPSS-V25 (IBM, Armonk, NY, USA) using ANOVA and Tukey post-hoc test, where the significance was at P value less than 0.05.

## Results

### Planktonic and biofilm susceptibility testing

Results revealed that CRPA-45 was resistant to all tested antibiotics. Whereas CRPA-47 was resistant to meropenem with intermediate susceptibility to both ceftazidime and gentamicin, while it showed sensitivity to only colistin. The tested *P. aeruginosa* isolates exhibited the highest susceptibility to colistin either in case of planktonic state or biofilms and that was indicated by the lowest recorded MIC, MBC, MBIC, and MBBC values compared to the other tested antibiotics. On the other hand, the least susceptibility was observed following treatment with ceftazidime. Also, an apparent elevation in the recorded concentrations of both the MBIC and MBBC compared to that of MIC and MBC by values of 4- and eightfold following treatment of both isolates with colistin and ceftazidime, respectively. A fourfold increase was also observed in the MBIC compared to MIC post-treatment of both isolates with gentamicin and meropenem. Whereas the elevation in the MBBC compared to MBC was in the order of 8- and 16-fold increase following treatment with gentamicin and meropenem for both isolates, respectively (Table 1).

**Table 1:** Antimicrobial susceptibility profiles against planktonic cells and biofilms of *P. aeruginosa* clinical isolates

Isolate	Antimicrobial agent	MIC (µg/ml)	Susceptibility	MBC (µg/ml)	MBIC (µg/ml)	MBBC (µg/ml)
CRPA-45	Ceftazidime	32	R	64	256	512
	Colistin	4	R	8	16	32
	Gentamicin	16	R	32	64	256
	Meropenem	16	R	32	64	512
CRPA-47	Ceftazidime	16	I	32	128	256
	Colistin	2	S	4	8	16
	Gentamicin	8	I	16	32	128
	Meropenem	8	R	16	32	256

*MIC* minimum inhibitory concentration, *R* resistant, *I* intermediate, *S* sensitive, *MBC* minimum bactericidal concentration, *MBIC* minimum biofilm inhibitory concentration, *MBBC* minimum biofilm bactericidal concentration

### Anti-biofilm of a single antibiotic

**Fig. 1:**
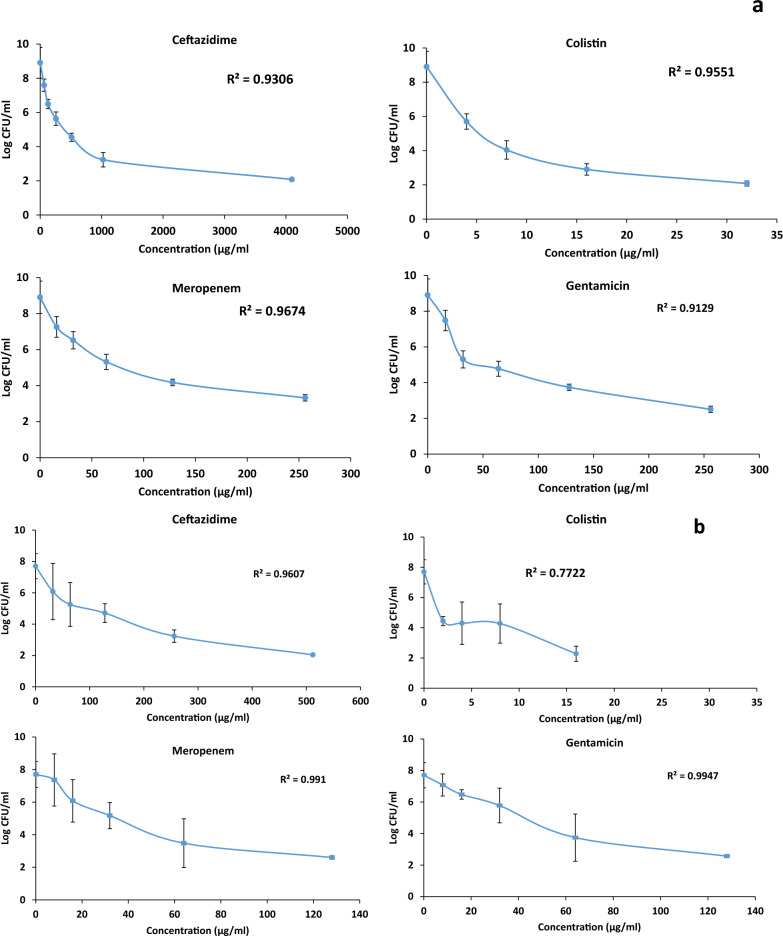
The total bacterial burden of *P. aeruginosa* isolates, **a**: CRPA-45 and **b**: CRPA-47, post biofilm treatment with different concentrations of antibiotics

Recorded data concerning the anti-biofilm of the single agent was used to calculate the EC_50_, area under the curve (AUC), and Hill factor of each antibiotic. Data were presented in a sigmoidal inhibitory Emax model. The sigmoidal Emax exhibited close-fitting to the data, where the recorded R^2^ value was in the range between 0.77 and 0.99 as shown in Fig. 1. Assessment of the anti-biofilm potential of each antibiotic using different concentrations equivalent to 0.25, 0.5, 1, 2, 4, 16, 32, and 64 times MBIC revealed statistically significant superior anti-biofilm potential in the case of colistin followed by gentamicin and meropenem, while the least anti-biofilm activity was observed post-treatment with ceftazidime (p < 0.05). The EC_50_ and AUC were determined for each antibiotic. Hill factor values lying in a range between 0.8 and 0.97 reveal a strong correlation between the concentrations of the tested antimicrobials and their subsequent anti-biofilm potentials (Table 2).

**Table 2:** Estimation of different parameters in the pharmacodynamic modeling of the anti-biofilm potential of a single antibiotic against *P. aeruginosa* isolates

Isolate	CRPA-45			CRPA-47		
Antimicrobial agent	EC_50_	AUC	Hill factor	EC_50_	AUC	Hill factor
Ceftazidime	2163.5	12675.2	0.78	350	2205.6	0.92
Colistin	22.4	120.4	0.84	15.2	70.4	0.84
Gentamicin	160.3	1256.5	0.87	91.5	670.8	0.97
Meropenem	202.5	1508.7	0.89	84.4	643.3	0.93

*EC*_*50*_ Effective concentration of the antibiotic that causes 50% of the maximum antibacterial effect, *AUC* area under the curve

### Fractional inhibitory concentration index

MICs of the tested antibiotics on planktonic cells alone and in combination were utilized to calculate the FICI. Synergism was observed as the calculated FICI was either less than 0.5 or equal to 0.5 following treatment of planktonic cells with antibiotic combinations of Gentamicin/Meropenem and Ceftazidime/Colistin, respectively (Table 3).

**Table 3:** MIC of the tested antibiotics on planktonic cells post single and combined treatments

Antimicrobial agent	MIC (µg/ml)	
	CRPA-45	CRPA-47
Ceftazidime	32	16
Colistin	4	2
Gentamicin	16	8
Meropenem	16	8
Ceftazidime in combination with colistin	8	4
Colistin in combination with ceftazidime	1	0.5
Gentamicin in combination with meropenem	2	1
Meropenem in combination with gentamicin	2	1
*FICI Ceftazidime/Colistin	0.5	0.5
*FICI Gentamicin/Meropenem	0.25	0.25

^*^FICI: Fractional inhibitory concentration index indicates synergism at a value less than or equal to 0.5

### Anti-biofilm of antibiotic combinations and pharmacodynamic modeling

**Fig. 2:**
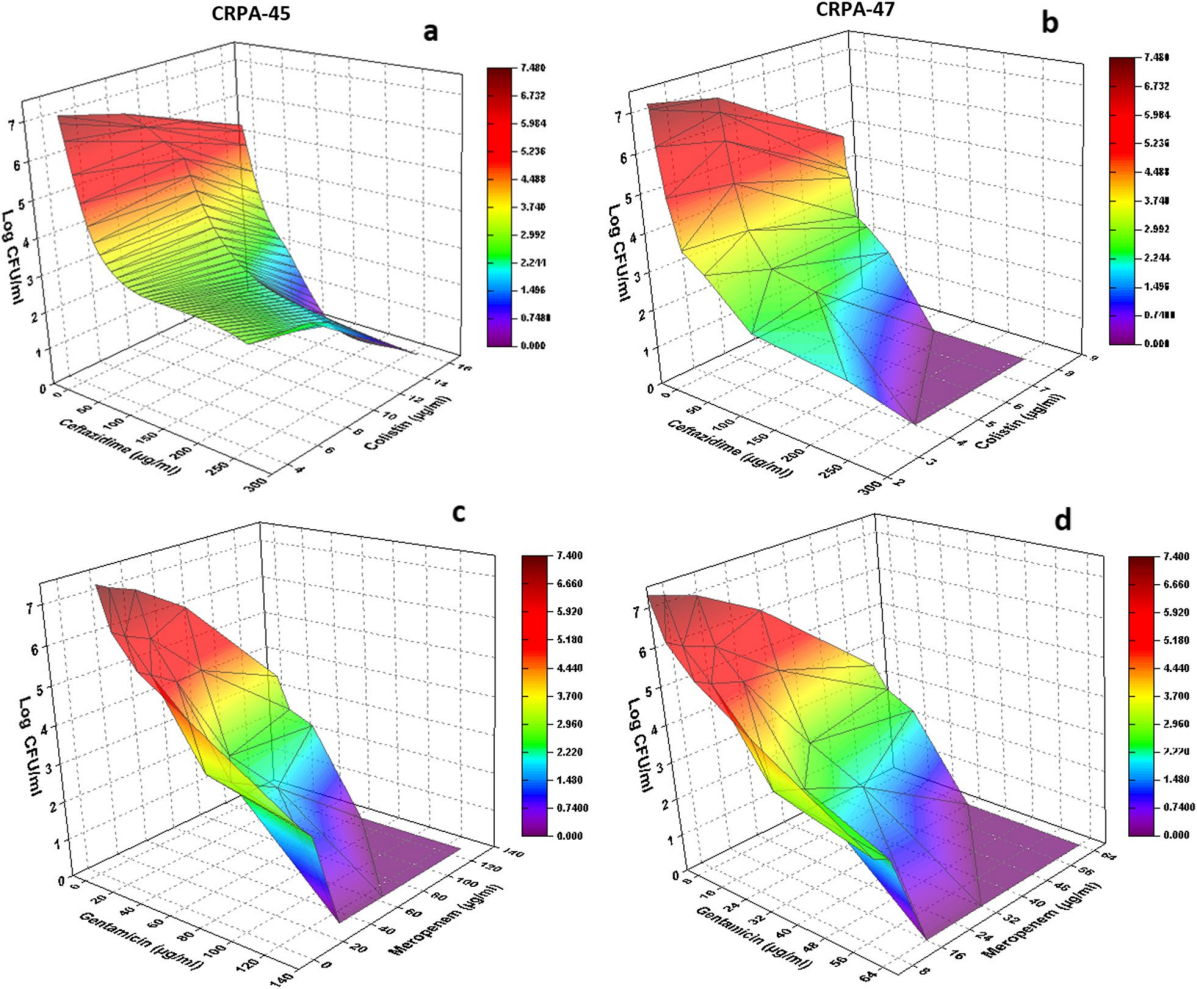
The detected bioburden level post-treatment of bacterial biofilms (CRPA-45 and CRPA-47) with variable concentrations of ceftazidime/colistin (**a** and **b**) and gentamicin/meropenem combinations (**c** and **d**)

The colored three-dimensional response surface plot in Fig. 2 revealed that synergism was observed along with increasing the concentrations of the tested antibiotics in combination with the highest synergism following the combination between colistin (16 µg/ml) and ceftazidime (64 µg/ml). However, increasing the concentration of ceftazidime to 128 and 256 µg/ml in combination with 16 µg/ml colistin was accompanied by complete inhibition of the biofilm in CRPA-45 (Fig. 2a). A similar pattern was observed in CRPA-47, where the maximum synergism was observed following treatment with 8 µg/ml colistin and 64 µg/ml ceftazidime till reaching 100% biofilm inhibitory potentials post-exposure to 8 µg/ml colistin in combination with either 128 or 256 µg/ml ceftazidime (Fig. 2b).

Results showed significantly (p < 0.05) higher anti-biofilm activity following treatment with gentamicin/meropenem combination as compared to that of ceftazidime/colistin combination. Also, the combination of gentamicin (128 µg/ml) and meropenem (23 or 64 µg/ml) exhibited total biofilm inhibition. A comparable observation was recorded when 128 µg/ml meropenem was combined with 64 or 128 µg/ml gentamicin in the case of CRPA-45 (Fig. 2c). Regarding CRPA-47, lower concentrations of both gentamicin (64 µg/ml) and meropenem (16 or 32 µg/ml) exerted a similar effect. Moreover, meropenem (64 µg/ml) when combined with either 32 or 64 µg/ml gentamicin resulted in complete inhibition of the preformed biofilms (Fig. 2d).

**Fig. 3:**
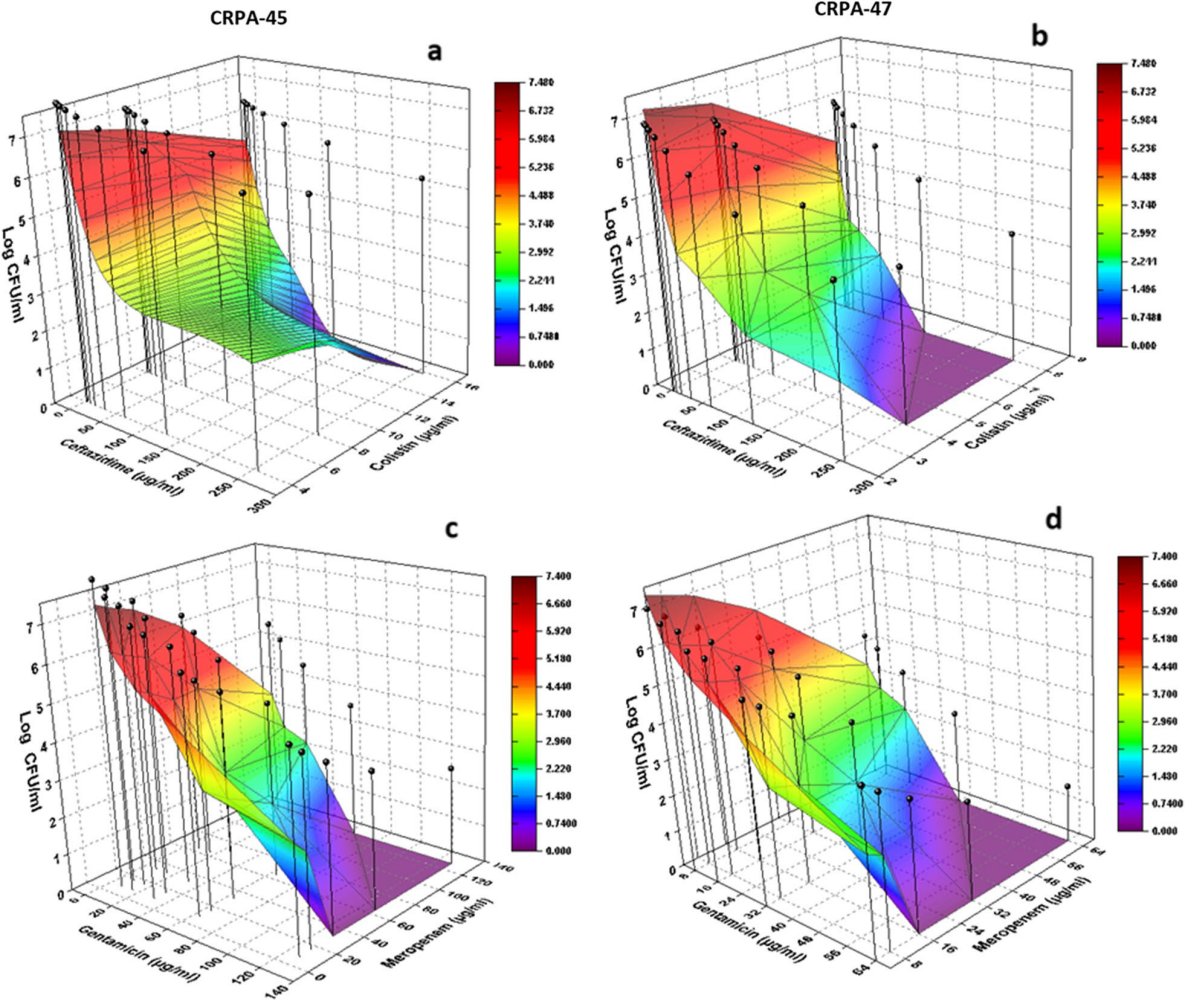
Graphical three-dimensional presentation comparing the observed (colored mesh) and the calculated simulated (vertical bars) anti-biofilm potentials of variable concentrations of ceftazidime/colistin (**a** and **b**) and gentamicin/meropenem (**c** and **d**) combinations. Results showed a lower bacterial burden (higher antibacterial potential) in the case of the observed data as compared to the simulated one under an assumption of null interaction between the combined antibiotics

In the same context, results revealed great similarity in pattern between the observed synergism detected following treatment with different antibiotic combinations as compared to that obtained via the calculated pharmacodynamic modeling (Fig. 3). Regarding the comparison between the results of the in vitro observation and that of the calculated pharmacodynamic modeling, Fig. 3 showed that the bacterial bioburden retrieved following treatment with different concentrations of antibiotics in combination (as indicated in the colored mesh) exerted higher observed anti-biofilm activity as compared to the simulated calculated anti-biofilm profile (black vertical bars) at all the tested concentrations of different antibiotic combinations.

## Discussion

*P. aeruginosa* is widely regarded as the most dangerous and typical biofilm-forming pathogen in humans [19]. *P. aeruginosa* biofilms could seriously hinder its eradication during antibiotic therapy and stimulate recurrent infections [20]. Moreover, the increasing frequency of *P. aeruginosa* resistance to many antibiotics especially to carbapenems is another major challenge, where it negatively influences the antibiotic treatment efficiency either alone or in combination [4].

Investigating the impact of antimicrobial combinations attracted the attention of many researchers in the last decade due to limited treatment options as a result of the increased prevalence of resistant organisms as well as another scope for reducing the toxicity of the antimicrobials [21]. Despite that different methods could be applied in vitro to estimate the efficacy of the combined antibiotics, the obtained findings might not exhibit remarkable benefit in expecting the clinical consequences of these combinations compared to the pharmacodynamic and in vivo models in addition to clinical studies [16]. Consequently, the present study assessed the in vitro anti-biofilm potentials of different antibiotic combinations compared to that obtained using mathematical pharmacodynamic modeling against CRPA clinical isolates, to establish an evidence-based rationale for the selection of antibiotic combinations.

Antimicrobial susceptibility usually evaluates the effectiveness of antibiotics against planktonic microorganisms; however, this is not always the current situation in case of infection with *P. aeruginosa* due to biofilm formation. Thus, the present study estimates the MBIC and MBBC along with the evaluation of the MIC and MBC, where the determination of the antibiotic’s MBIC is essential as it reflects its role in the treatment of biofilm-forming *P. aeruginosa* infections [20]. Also, the observed resistance to different antibiotics in the present study is an essential issue to highlight, where several antibiotic resistance mechanisms might be engaged in this resistance. For example, β-lactamase overproduction is the main mechanism responsible for resistance to ceftazidime [22]. Whereas the most common strategies for resistance to colistin are the alterations of the bacterial outer membrane via modifying the structure of its lipopolysaccharide and reducing its negative charge, in addition to overexpression of the efflux-pump regulators [23]. Resistance to carbapenems is usually multifactorial where it includes the acquisition of carbapenemase encoding genes through horizontal gene transfer, downregulation of the porin (OprD) for carbapenem as well as overexpression of *mexAB-oprM* efflux pump [24]. It was also reported that *P. aeruginosa* resistance to aminoglycosides is correlated to the production of aminoglycoside-modifying enzymes as well as efflux mechanisms of resistance [25].

The currently recorded low rate of reduction of the bacterial bioburden although the biofilms were treated with increasing concentrations of the tested antibiotics is mainly due to that bacterial biofilms are extremely resistant to antibiotics compared to planktonic cells. It was reported that most antibiotics such as colistin could only reduce the bacterial bioburden in the biofilms without eradicating it. The biofilm formation enhances the resistance to antibiotics due to its mucoid structure, especially in the case of *P. aeruginosa*. Additionally, the low metabolic activity of bacteria in biofilms as well as the inadequate oxygen supply renders them more resistant to antimicrobials. Moreover, subjecting the bacterial biofilms to sub-lethal concentrations of antibiotics was accompanied by higher rates of the transfer of antibiotic resistance genes as well as the development of persisters [26].

Checkerboard assay and E-test-based methods are the most commonly applied methods and are also considered promising methods to evaluate the effectiveness of antibiotics in combinations. [21]. Thus, the checkerboard assay was applied in the present study as an indicator of the antibiotic combination profile.

Unfortunately, antimicrobial resistance is worsening. There is evidence that the proportion of Gram-negative organisms that are resistant to commonly used antibiotics is increasing even to antibiotics that are considered as rescue therapy, such as colistin [27]. Colistin is also regarded as a drug that contributes effectively to the treatment of CRPA [28]. Similar to the current findings, a synergism was reported upon a combination between colistin and ceftazidime in the case of multi-antibiotic-resistant *P. aeruginosa*, although this study was conducted only on planktonic cells [29]. It was also demonstrated that the combination of β-lactam (ceftazidime) and polymyxin (colistin) antibiotics could reduce the MICs of the tested antibiotics against *P. aeruginosa* isolates [30].

Recorded results demonstrated that the anti-biofilm potentials of ceftazidime/colistin in combination are proportional to increasing the concentration of the tested antibiotics, especially in the case of ceftazidime, where higher ceftazidime concentrations were accompanied by complete inhibition of *P. aeruginosa* biofilms. In agreement, a recent study reported elevated anti-biofilm potentials following continuous infusion of elevated concentrations of ceftazidime (40 mg/L) when combined with colistin compared to that observed in case of infusion using ceftazidime at only a concentration of 4 mg/L in combination with colistin against *P. aeruginosa* biofilm-associated infections. That could be attributed to that elevated ceftazidime concentrations might be accompanied by a higher degree of ceftazidime dispersion in the bacterial biofilms thus allowing its interaction with the bacterial subpopulations with different metabolic activity in the biofilms and overcoming the antibiotic tolerance [22].

The mechanisms implicated in the synergism developed due to the colistin and ceftazidime combination aren’t fully recognized. A study demonstrated the effectiveness of colistin against the metabolically less active bacteria which are deeply embedded in the biofilms [31]. On the contrary, beta-lactams (ceftazidime) could predominantly kill bacteria in the external surfaces of the biofilms, where the existing bacteria exhibited higher metabolic activity [32]. Colistin in combination with ceftazidime resulted in more effective anti-biofilm potential, where colistin disrupts the integrity of the biofilms and facilitates the accessibility of the beta-lactam antibiotic to deeper bacterial populations in the biofilms. Colistin can also increase the cellular permeability of antibiotics. That is accompanied by the well-known cell wall inhibitory potentials of beta-lactams [22]. Consequently, the effectiveness of ceftazidime in combination with colistin on several layers of the biofilm in addition to variable cellular targets could potentially account for the observed synergism following their combination. It is essential to point out that the targeted antibiotic combinations could be effective when synergistic antibiotics were applied based on their pharmacodynamic properties. A recent case report study demonstrated treatment failure of a patient suffering from *P. aeruginosa* catheter*-*associated infection using colistin either alone or in combination with ceftazidime as the patient developed nephrotoxicity signs following colistin treatment. In an attempt to find a proper treatment, the patient was treated with meropenem, gentamicin, and rifampicin in combination based on the obtained successful indications of the pharmacodynamics of these antibiotics in combination*.* On the fifth day of treatment with such a triple antibiotic combination, a negative urine culture was observed despite that these antibiotics were ineffective in vitro [33]. On the other side, another study reported that colistin toxicity could be reduced via its administration over extended time intervals leading to less tissue accumulation, with a subsequent reduction in its adverse effects [29].

Similar to the current findings, a synergism was observed following the treatment of *P. aeruginosa* clinical isolates with imipenem and gentamicin in combination using checkerboard assay against planktonic cells [34]. Another study reported that the combination of meropenem (MIC ≤ 8 mg/L) with gentamicin resulted in a reduction in mortality, especially in patients with septic shock [28]. The currently recorded synergism between meropenem and gentamicin could be justified by that the combination of β-lactam (meropenem) and aminoglycoside (gentamicin) supports different mechanisms of bacterial killing. β-Lactam mediates the interference with the synthesis of vital cell wall components, which in turn facilitates the passage of aminoglycosides into the periplasmic space thus inhibiting the synthesis of the bacterial protein by binding to 30S ribosomes. However, it was also reported that this in vitro synergy appears to be variable with different β-lactam and aminoglycoside combinations [27].

Despite that, the two tested clinical isolates were categorized as moderate biofilm producers [8] but they showed variation in the extent of their response to the antibiotics in combination. That was apparent in that the combined antibiotics exhibited higher anti-biofilm inhibitory potentials against CRPA-47 isolate compared to that obtained in the case of CRPA-45. It is essential to point out that the variation in the ability of the bacterial cells to develop biofilms should be regarded as a factor contributing to variable anti-biofilm potentials of the tested antimicrobials. This is possibly due to that there is a correlation between variable expression levels of biofilm formation genes, which is associated to different abilities of biofilm production, and the bacterial response to antimicrobials [35].

The well-known discrepancy between bacterial growth in vitro* and *in vivo is another important issue to be highlighted in the current study, where bacterial multiplication in vitro occurs at a higher rate compared to in vivo. Therefore, stronger competition for nutrients could result in enhanced synthesis of antibacterial cellular targets, leading to an elevation in the in vitro antimicrobial susceptibility [36]. That could justify the recorded higher in vitro observed anti-biofilm potentials of the tested antibiotic combinations compared to that obtained via the simulated pharmacodynamic modeling. On the other side, in vitro pharmacodynamic modeling permits the estimation of the in vivo bacterial multiplication in addition to comparing several dosage schedules either in the case of a single antibiotic or for variable antibiotic combinations. Consequently, this model could effectively contribute to dose optimization as well as successful antibiotic combinations for achieving a respectable clinical outcome using currently available antibiotics [36].

As far as we know, the current study is the first report where both the in vitro experiments, as well as the mathematical pharmacodynamic modeling, have been employed to explore the anti-biofilm potentials of ceftazidime/colistin versus gentamicin/meropenem combinations against *P. aeruginosa* biofilm. This study represents an effective approach for the assessment of the antimicrobial activity against inherently resistant bacterial biofilm with an evidence-based selection of appropriate antibiotic therapy.

## Conclusions

The current study explored the anti-biofilm synergistic potentials of ceftazidime/colistin and gentamicin/meropenem combinations against carbapenem-resistant biofilm-forming *P. aeruginosa* clinical isolates either in vitro or via the mathematical pharmacodynamic modeling. The higher anti-biofilm activity was observed post-treatment with gentamicin/meropenem as compared to that with the ceftazidime/colistin combination. The study also shed light on the application of mathematical pharmacodynamic modeling in investigating the efficacy of new antibiotic combinations as a guide for antibiotic therapy rather than using laborious and time-consuming laboratory methods, especially in biofilm-associated infections.

### Study limitations

A limitation of the present study is assessing the effect of anti-biofilm potentials of antibiotic combinations on two *P. aeruginosa* isolates. Therefore, future studies will target examining the impact of variable combinations on different clinical isolates.

## Data Availability

All data generated or analyzed during this study are included in this published article.

## Abbreviations

CRPA: Carbapenem-resistant *Pseudomonas aeruginosa*
FICI: Fractional inhibitory concentration index
MDR-PA: Multiple drug-resistant *P. aeruginosa*
TSB: Tryptic soy broth
MHB: Mueller Hinton broth
CFU: Colony forming unit
CLSI: Clinical and laboratory standard institute
MIC: Minimum inhibitory concentration
MBC: Minimum bactericidal concentration
MBIC: Minimum biofilm inhibitory concentration
MBBC: Minimum biofilm bactericidal concentration
MHA: Mueller Hinton agar
EC_50_: Effective concentration of the antimicrobial agent that caused 50% of the maximum effect
AUC: Area under the curve
